# Electroacupuncture Promotes Neuroplasticity of Central Auditory Pathway: An Auditory Evoked Potentials Study

**DOI:** 10.1155/2022/6855775

**Published:** 2022-11-21

**Authors:** Chia-Hao Chang, Chia-Der Lin, Ching-Liang Hsieh

**Affiliations:** ^1^Graduate Institute of Acupuncture Science, China Medical University, Taichung, Taiwan; ^2^Department of Chinese Medicine, China Medical University Hospital, Taichung, Taiwan; ^3^Division of Otorhinolaryngology, China Medical University Hospital, Taipei Branch, Taipei, Taiwan; ^4^Department of Otorhinolaryngology, China Medical University Hospital, Taichung, Taiwan; ^5^School of Medicine, China Medical University, Taichung, Taiwan; ^6^Chinese Medicine Research Center, China Medical University, Taichung, Taiwan

## Abstract

Our previous studies found that electroacupuncture at the right Zhongzhu acupoint (TE3) can enhance auditory recovery in rats with noise-induced hearing loss. Here, we investigated the changes in auditory brainstem response (ABR) and long late latency (LLR) evoked potential to explain the mechanisms of electroacupuncture at TE3. The auditory evoked potentials were recorded, including ABR and LLR, at baseline and on day 3 (D3), D5, and D8 after baseline. The 2-Hz electroacupuncture at the right TE3 was applied on D3, D4, and D5 in the electroacupuncture group but not in the control group. In ABR, compared with the control group, the latency shift of waves I (0.298 ± 0.033 vs −0.045 ± 0.057 ms), III (0.718 ± 0.038 vs −0.163 ± 0.130 ms), and V (1.160 ± 0.082 vs −0.207 ± 0.138 ms) on D3 (all *p*  <  0.01) and of wave V (0.616 ± 0.433 vs −0.352 ± 0.209 ms, *p*  <  0.05) on D5 was greater in the electroacupuncture group than that in the control group. Moreover, the interpeak latency shift of I–III (0.420 ± 0.041 vs −0.118 ± 0.177 ms) and I–V (0.863 ± 0.088 vs −0.162 ± 0.156 ms) on D3 (both *p*  <  0.05) and of III–V (0.342 ± 0.193 vs −0.190 ± 0.110 ms) and I–V (0.540 ± 0.352 vs −0.343 ± 0.184 ms) on D5 (both *p*  <  0.05) was greater in the electroacupuncture group than that in the control group. In LLR, the latency shift of P0 was greater in the electroacupuncture group than in the control group on D3 (3.956 ± 2.975 vs −1.178 ± 1.358 ms, *p*  <  0.01) and D5 (2.200 ± 1.889 vs −0.311 ± 1.078 ms, *p*  <  0.05). These findings indicate that electroacupuncture at the right TE3 can modulate the neuroplasticity of the central auditory pathway, including the brain stem and the primary and secondary auditory cortex.

## 1. Introduction

Tinnitus is generated from peripheral damage, and it mainly affects the central auditory system, including the primary auditory cortex and nonauditory higher centers, through changes in cortical excitation and inhibition [1]. According to the auditory plasticity theory of tinnitus, cochlear damage can promote neural activity of the central auditory pathway, which connects the cochlear nucleus to the auditory cortex [2, 3]. A decrease in the sensory input from the cochlea can cause a compensatory enhancement of the central gain, leading to tinnitus and hyperacusis [4, 5]. Therefore, neuroplasticity plays a critical role in the development of tinnitus.

Auditory brainstem response (ABR) measures the neuronal activity of the auditory pathway from the cochlea to the auditory cortex. An ABR waveform has seven prominent wave peaks occurring within 10 ms of the auditory stimulus; of them, waves I, III, and V are clearly generated [6, 7] and are used clinically to assess the cochlea, auditory nerve, and brainstem function [8]. The auditory middle latency response (MLR) is produced between 10 and 80 ms after the auditory stimulus onset and has multiple generators, mainly from the thalamocortical pathways. The first trough of the MLR is known as Na, which is followed by three peaks—Pa, Nb, and Pb [9]. Auditory late latency response (LLR) is generated at 60–80 ms, 90–100 ms, and 100–169 ms, respectively, after auditory stimulus, with the peaks of P1, N1, and P2; LLR has multiple generators, including primary and secondary auditory cortices [10]. Because both MLR and LLR were generated 10 ms after auditory stimulation onset, and there is a partial overlap between them. In addition, the origin of both MLR and LLR is still unknown. Therefore, in the present study, we considered the entire waveform from 10 ms to 100 ms, collectively naming it LLR [11]. Acupuncture therapy originated more than 2500 years ago in ancient China and is now widely used worldwide, including in Western countries [12]. Acupuncture therapy is mentioned in the National Institutes of Health (NIH) conference and is recorded in the Journal of the American Medical Association (JAMA) [13]. Acupuncture is based on the meridian theory described in the Huang Di Nei Jing (The Yellow Emperor's Classic of Internal Medicine). Electroacupuncture is a modern variation that uses low current and frequency stimulation between stainless steel needles inserted into two acupoints. Its therapeutic mechanism is the modulation of the peripheral and central nerve pathways based on neurophysiological behavior [14]. The Zhongzhu (TE3) acupoint belongs to the Sanjiao meridian, which runs around the ear; therefore, acupuncture at TE3 can treat ear diseases such as tinnitus and deafness in traditional Chinese medicine (TCM) [15], but its curative effect and mechanism remain unclear.

We previously reported that electroacupuncture at the right TE3 can enhance auditory recovery and reduce the loss of spiral ganglion neurons in rats with noise-induced hearing loss [16]. Therefore, in the present study, we expanded on our previous study by investigating the changes in ABR and LLR recordings to obtain insights into the central mechanism of electroacupuncture at right TE3.

## 2. Materials and Methods

### 2.1. Animals

Twelve male 8–12-week-old Sprague Dawley (SD) rats were purchased from BioLASCO, Taiwan, and bred in the Animal Center of China Medical University. Two rats were placed together in a cage under regular 12 h light/12 h dark conditions. The room temperature was maintained between 22°C and 24°C and humidity between 50% and 70% by using an air conditioner. Adequate food and water were provided to the rats. All experimental procedures were performed in accordance with the guidelines of the Animal Experimentation Ethics Committee (IACUC-2017-068).

### 2.2. ABR and LLR Recordings

First, the rats were anesthetized through intraperitoneal injection with Zoletil 50 and xylazine, and then placed on a thermal blanket. Their body temperature was maintained at 37°C. An earphone (Etymotic ER-2A, Etymotic Research Inc., Wlk Grove Village, IL, USA) was placed into the left external auditory canal. A recording subcutaneous needle electrode was placed on the ipsilateral temporal region just above the upper edge of the ear, and a reference subcutaneous needle electrode was placed on a similar location on the contralateral side. Another subcutaneous needle electrode was then placed on the midpoint of the rat head; this served as a ground electrode. The impedance of all the electrodes was 1–3 kΩ. Finally, these electrodes were connected to an auditory evoked potential system (SmartEP, Intelligent Hearing System, Miami, FL, USA) for the ABR and LLR recordings.

**Figure 1:**
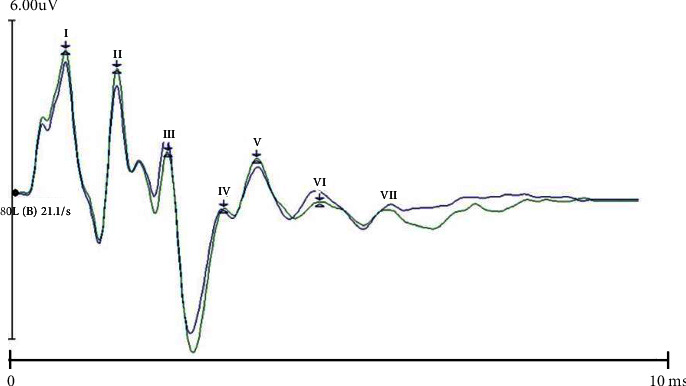
Series of auditory brainstem response (ABR). Seven waves of I, II, III, IV, V, VI, and VII within 10 ms from auditory stimulus onset in ABR. Waves I, III, and V were used for clinical analysis.

For ABR, the sound of the auditory stimulus was conducted by the process system (tone pipes). We used click sounds delivered at 80 dB nPL at a rate of 21.1 stimuli per second, and the bandpass filter was set between 100 Hz and 1500 Hz. A total of 1024 responses were averaged, with an analysis time of 12.8 ms. In each session, the trials were repeated three times, and two waveforms that were similar and reproducible were selected. The peak latency and interpeak latency of waves I, III, and V were measured (Figure 1).

**Figure 2:**
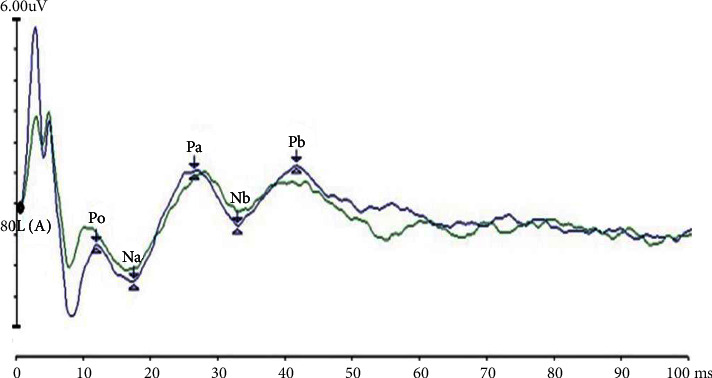
Series of auditory long latency response (LLR). A series positive–negative waves, namely P0, Na, Pa, Nb, and Pb, from 10 ms to 100 ms after auditory stimulus onset in LLR.

For LLR, the locations of electrodes and recording methods were similar to those in ABR. Rarefaction click sounds, 0.1 s in duration and delivered at 80 dB nHL and a rate of 7.1 stimuli per second, were delivered from the earphone. The bandpass filter was set between 10 and 1500 Hz. A total of 1024 responses were averaged, with an analysis time of 100 ms. The peak latencies of P0, Na, Pa, Nb, and Pb waves and the interpeak amplitudes of P0–Na, Na–Pa, Pa–Nb, and Nb–Pb were measured. In each session, the trials were repeated three times, and two waveforms that were similar and reproducible were selected (Figure 2).

### 2.3. Acupuncture Intervention

**Figure 3:**
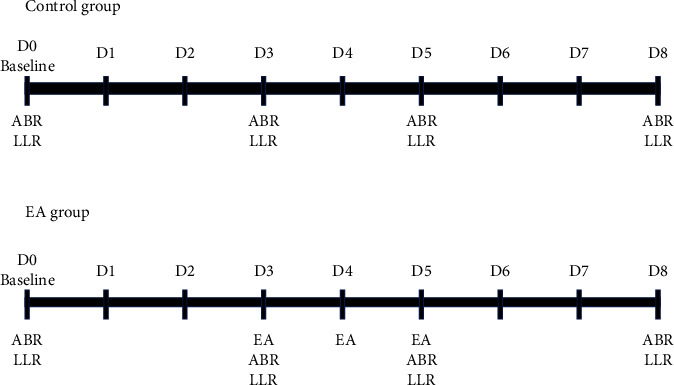
Experimental procedure. The experimental rats were divided into the control group without electroacupuncture (EA) intervention and the EA group with EA intervention. ABR: auditory brainstem response; LLR: long latency response; Baseline: baseline recordings; D1: first day after baseline recordings; D2: second day after baseline recordings; D3: third day after baseline recordings; D4: fourth day after baseline recordings; D5: fifth day after baseline recordings; D6: sixth day after baseline recordings; D7: seventh day after baseline recordings; D8: eighth day after baseline recordings; EA: electroacupuncture intervention.

A stainless steel needle (length 2.54 cm, gauge 34, Chian Huei Acupuncture Device, Taipei City, Taiwan) was inserted into the right TE3 in rats (under anesthesia: intraperitoneal zoletil 50 + xylazine), which is equivalent to the dorsal position of the foot between the fourth and the fifth metacarpal bones in humans; the other needle was inserted 0.5 cm proximal to the TE3. Next, the needles were connected to an electrical stimulation device (Trio 300, Japan). Electric stimuli were delivered for 3 consecutive days (D3, D4, and D5, Figure 3) and each group had six rats that were based on our previous study [17] in the electroacupuncture group with the following parameters: frequency, 2 Hz; wave width, 250 *μ*s; intensity, 2 mA; and duration, 1 h.

### 2.4. Study Design

Twelve rats were randomly divided into a control group (*n* = 6) and an electroacupuncture group (*n* = 6). In the control group (without electroacupuncture intervention), ABR and LLR were recorded at baseline and 3 days later (D3), D5, and D8 (Figure 3). In the electroacupuncture group, ABR and LLR were recorded at baseline; electroacupuncture was delivered on D3, D4, and D5; and ABR and LLR were recorded again on D3, D5, and D8. The order of the recordings was as follows: ABR, followed by LLR, and then ABR and LLR recordings following electroacupuncture (Figure 3).

### 2.5. Statistical Analysis

We collected the amplitude and latency of ABR and LLR waveforms in Control and electroacupuncture group We calculated the value of D3, D5, or D8 minus the baseline value as the shift value. Then, we calculate the mean and standard deviation (SD) (*n* = 6, in each group).

After descriptive statistics, first, we explored the changes in the ABR and LLR waveforms before and after electroacupuncture. We compared ABR and LLR waveforms, including amplitude and latency, between baseline and D3, baseline and D5, and baseline and D8 in each group. Next, we compared the waveform shift value of ABR and LLR between the two groups. All these analyses were performed using a mixed-effects model and the Tukey multiple comparisons test in the Prism program (version 9.3.1 for macOS, GraphPad software). *p*  <  0.05 was considered statistically significant.

## 3. Results

### 3.1. Electroacupuncture at the Right TE3 Altered ABR Latency

**Figure 4:**
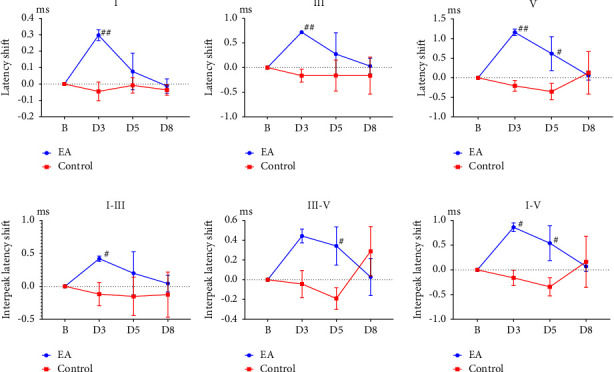
Effect of electroacupuncture on peak and interpeak latency shift of auditory brainstem response (ABR). EA: electroacupuncture group; control: control group; I: wave I latency shift of ABR; III: wave III latency shift of ABR; V: wave V latency shift of ABR; I–III: wave I–III interpeak latency shift of ABR; III–V: wave III–V interpeak latency shift of ABR; I–V: wave I–V interpeak latency shift of ABR; B: baseline; D3: third day after baseline recordings; D5: fifth day after baseline recordings; D8: eighth day after baseline recordings. ^#^*p* < 0.05, ^##^*p* < 0.05 compared with Control.

The wave I peak latency at baseline was not significantly different between the electroacupuncture and control groups (*p*  >  0.05; Table 1); it was significantly lower than the value on D3 (*p*  <  0.01; Table 1) but approximate to the value on D5 and on D8 in the electroacupuncture group (both *p*  >  0.05; Table 1). The peak latency shift of wave I in the electroacupuncture group was significantly greater than that in the control group value on D3 (*p*  <  0.01; Table 1; Figure 4), but the between-group differences were not significant on D5 and D8 (both *p*  >  0.05; Table 1; Figure 4).

The wave III peak latency at baseline was not significantly different between the electroacupuncture and control groups (*p*  >  0.05; Table 1); it was significantly lower than the value on D3 (*p*  <  0.001; Table 1) but was comparable to the values on D5 and on D8 (both *p*  >  0.05; Table 1) in the electroacupuncture group. The peak latency shift of wave III in the electroacupuncture group was significantly greater than that in the value on D3 (*p*  <  0.01; Table 1; Figure 4), but the between-group differences were not significant on D5 and D8 (both *p*  >  0.05; Table 1; Figure 4).

Wave V latency at baseline was not significantly different between the electroacupuncture and control groups (*p*  >  0.05; Table 1); it was significantly lower than the value on D3 (*p*  <  0.001; Table 1) but was comparable to the values of D5 and on D8 (both *p*  >  0.05; Table 1) in the electroacupuncture group. The latency shifts of wave V in the electroacupuncture group were significantly greater than that in the control group on D3 and D5 (*p*  <  0.01, *p*  <  0.05, respectively; Table 1; Figure 4), but the between-group differences were not significant on D8 (*p*  >  0.05; Table 1; Figure 4).

The I–III interpeak latency at baseline was not significantly different between the electroacupuncture and control groups (*p*  >  0.05; Table 1); it was significantly lower than that in the value on D3 (*p*  >  0.05; Table 1) but was comparable to the value on D8 (both *p*  >  0.05; Table 1) in the electroacupuncture group. The I–III interpeak latency shift in the electroacupuncture group was significantly greater than that in the control group (*p*  <  0.05; Table 1; Figure 4) on D3, but the between-group differences were not significant on D5 and D8 (both *p*  >  0.05; Table 1; Figure 4).

The III–V interpeak latency at baseline was not significantly different between the electroacupuncture and control groups (*p*  >  0.05; Table 1); it was significantly lower than that in the value on D3 (*p*  <  0.01; Table 1) but was comparable to the value on D5 and on D8 (both *p*  >  0.05; Table 1) in the electroacupuncture group. The III–V interpeak latency shift in the electroacupuncture group was significantly greater than that in the control group on D5 (*p*  <  0.05; Table 1; Figure 4), but the between-group differences were not significant on D3 and D8 (both *p*  >  0.05; Table 1; Figure 4).

The I–V interpeak latency at baseline was not significantly different between the electroacupuncture and control groups (*p*  >  0.05; Table 1); it was significantly lower than that in the value on D3 (*p*  <  0.01; Table 1) but was comparable to the value on D5 and on D8 (both *p*  >  0.05; Table 1) in the electroacupuncture group. The I–V interpeak latency shifts in the electroacupuncture group were significantly greater than that in the control group on D3 and D5 (*p*  <  0.05; Table 1; Figure 4), but the between-group differences were not significant on D8 (*p*  >  0.05; Table 1; Figure 4).

### 3.2. Electroacupuncture at the Right TE3 Altered LLR Peak Latency

**Figure 5:**
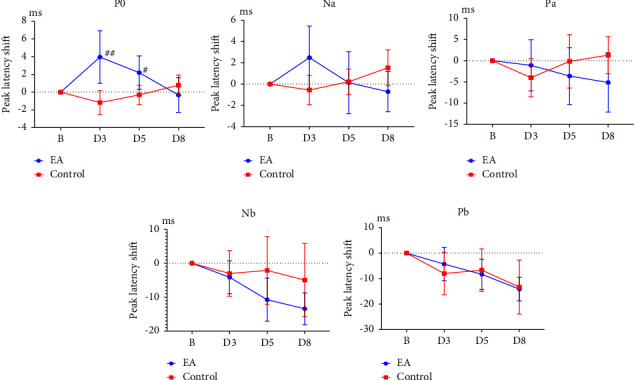
Effect of electroacupuncture on latency shift of auditory long latency response (LLR). EA: electroacupuncture group; control: control group; P0: wave P0 latency shift of LLR; Na: wave Na latency shift of LLR; Pa: wave Pa latency shift of LLR; Nb: wave Nb latency shift of LLR; Pb: wave Pb latency shift of LLR; B: baseline; D3: third day after baseline recordings; D5: fifth day after baseline recordings; D8: eighth day after baseline recordings. ^#^*p* < 0.05, ^##^*p* < 0.01 compared with control.

P0 latency at baseline was not significantly different between the electroacupuncture and control groups (*p*  >  0.05; Table 2); it was less than the value on D3 and D5 (both *p*  <  0.05; Table 2) but was similar to the value on D8 (*p*  >  0.05; Table 2) in the electroacupuncture group. The latency shifts of P0 in the electroacupuncture group on D3 and D5 were greater than in the control group on D3 and D5 (*p*  <  0.01, *p*  <  0.05, respectively; Table 2; Figure 5).

Na latency at baseline was not significantly different between the electroacupuncture and control groups (*p*  >  0.05; Table 2); it was similar to the value on D3, D5, and D8 in the electroacupuncture group (all *p*  >  0.05; Table 2). The Na latency shift was not significantly different between the two groups on D3, D5, or D8 (all *p*  >  0.05; Table 2; Figure 5).

Pa latency at baseline was not significantly different between the electroacupuncture and control groups (*p*  >  0.05; Table 2); it was similar to the value on D3, D5, and D8 in the electroacupuncture group (all *p*  >  0.05; Table 2). The Pa latency shift was not significantly different between the two groups on D3, D5, or D8 (all *p*  >  0.05; Table 2; Figure 5).

Nb latency at baseline was not significantly different between the electroacupuncture and control groups (*p*  >  0.05; Table 2); it was greater than that in the value on D5 and D8 (*p*  <  0.01, *p*  <  0.001, respectively, Table 2) but was comparable to the value on D3 (*p*  >  0.05; Table 2) in the electroacupuncture group. The Nb latency shift was not significantly different between the two groups on D3, D5, or D8 (all *p*  >  0.05; Table 2; Figure 5).

Pb latency at baseline was not significantly different between the electroacupuncture and control groups (*p*  >  0.05; Table 2); it was significantly greater than that in the value on D5 and D8 (*p*  <  0.05, *p*  <  0.0001, respectively, Table 2) but was comparable to the value on D3 (*p*  >  0.05; Table 2) in the electroacupuncture group. The Pb latency shift was not significantly different between the two groups on D3, D5, and D8 (all *p*  >  0.05; Table 2; Figure 5).

### 3.3. Electroacupuncture at the Right TE3 Altered LLR Interpeak Amplitude

The P0–Na amplitude was not significantly different within the electroacupuncture group on D3, D5, and D8 compared with baseline (all *p*  >  0.05; Table 2) or between the electroacupuncture and control groups on D3, D5, and D8 (all *p*  >  0.05; Table 2).

The Na–Pa amplitude in the electroacupuncture group was greater than that in the value at baseline on D5 (*p*  <  0.05; Table 2), and was less than that in the value at baseline on D8 (*p*  <  0.05; Table 2) but was comparable to baseline on D3 (*p*  >  0.05; Table 2); no significant difference was noted between the electroacupuncture and control groups at baseline, D3, D5, and D8 (all *p*  >  0.05; Table 2).

The Pa–Nb amplitude was not significantly different within the electroacupuncture group on D3, D5, and D8 compared with baseline or between the electroacupuncture and control groups on D3, D5, and D8 (all *p*  >  0.05; Table 2).

The Nb–Pb amplitude was not significantly different within the electroacupuncture group on D3, D5, and D8 compared with baseline or between the electroacupuncture and control groups on D3, D5, and D8 (all *p*  >  0.05; Table 2).

## 4. Discussion

Our results revealed that electroacupuncture at right TE3 induced the peak latency shift of waves I, III, and V on D3 and of wave V on D5 as well as I–III and I–V interpeak latency shifts on D3 and the III–V interpeak latency shift on D5. ABR is generated using an auditory stimulus to induce the evoked potential of the brain stem, and its main neurophysiological function is to measure brainstem function and integrity [8]. In humans, ABR is primarily generated by the cochlear nuclear complex, medial olivary nucleus, dorsal lemniscal nucleus, and inferior colliculus, and the auditory information is processed from the cochlear nucleus through the superior olivary complex, medial lemniscus, and finally reaches the inferior colliculus [18]. The ABR can also reflect the neural activity of the auditory pathway from the cochlea to the auditory cortex [6]. Wave I is generated from the auditory nerve, wave III from the superior olivary complex, and wave V from the inferior colliculus [8]. Therefore, electroacupuncture at the right TE3 can affect the physiological function of the auditory pathway, at least from the cochlear nucleus to the inferior colliculus. This result is similar to that of our previous study, which reported that electroacupuncture at the right TE3 can promote auditory recovery and also can protect spiral ganglion neuronal cell damage in rats with noise-induced hearing loss [16]. The results are also consistent with the notion that cochlear damage may cause the downregulation or upregulation of the central auditory pathway to compensate for the amount of neural activity from the injured cochlea [2].

Our results also indicated that the latency shift of P0 in the electroacupuncture group was greater than that in the control group on D3 and D5; the latency shift of Nb and Pb in the electroacupuncture group was lower at baseline than that on D5 and D8; the Na–Pa amplitude in the electroacupuncture group was greater and was lower at baseline than that on D5 and D8, respectively. ABR was generated within 10 ms after auditory stimulus onset, whereas a series of MLR evoked potentials in the scalp occurred between 10 and 50 ms [7]. Neves et al. reported that MLR occurs between 10 and 80 ms after the onset of auditory stimulation; it has multiple origins, mainly the thalamocortical pathway [9]. LLR is a series of positive–negative peak potentials generated 60 ms after auditory stimulation onset. It has multiple origins, including primary and secondary auditory cortices [10]. In the present study, we used LLR to consider evoked potentials generated between 10 and 100 ms in the rat scalp. Our results revealed that the peak latency of P0, Na, Pa, Nb, and Pb was 14.867, 20.444, 37.533, 55.311, and 69.556 ms, respectively; in the control group, these values fell between 10 and 100 ms. Therefore, electroacupuncture at the right TE3 could cause P0, Nb, and Pb latency shift and the interpeak amplitude shift of Na–Pa, thus possibly inducing central auditory neuroplasticity. Acupuncture can relieve pain and treat neurodegenerative diseases possibly through effects on neuroplasticity, including dendrite remodeling, long-term potentiation, and neurogenesis [19]. A report analyzing magnetic resonance imaging studies concluded that acupuncture can reorganize motor-, sensory-, language-, and cognition-related networks in poststroke patients, suggesting that acupuncture treatment can modulate neuroplasticity in terms of the function and structure of the central nervous system after stroke [20]. Taken together, these results suggest that electroacupuncture at the right TE3 can promote neuroplasticity from the brain stem to the primary and secondary auditory cortex and is beneficial for functional recovery after damage. In TCM, the TE3 acupoint is located on the hand and belongs to the triple energizer meridian of the hand connected to the ear. Therefore, acupuncture at TE3 can treat ear diseases, such as deafness and tinnitus [15]. In addition, the flowing of meridian qi is similar to a circle with no end, therefore, acupuncture on one side treats diseases on the contralateral side [21].

One needs to be explained in our results showed both ABR and LLR patterns seem changes in the control and electroacupuncture groups on day 8 in the III–V of Figure 4, and in the P0, Na, Pa and Nb of Figure 5. However, there are not significant differences between the control and electroacupuncture group (Supplement 1). Based on these results, we speculated that the effects of EA at TE3 on ABR and LLR could not be maintained until the third day (D8) after EA treatment. The effect of EA returned to baseline that was similar to the control group. Because the present study used healthy rat the results whether it is similar to rat with noise-induced hearing loss or with cochlear artery ischemia needs to be further studied in the future.

The following are some limitations of the present study: (1) Although electroacupuncture at TE3 can cause latency shift of P0, Nb, and Pb, the generation of P0, Nb, and Pb remains unknown. (2) Our experiments were performed in healthy rats, and the results may not be generalizable to humans or diseased animals, thus warranting further study. (3) The present study lacks structural plasticity in terms of microarchitecture including dendritic complexity and spine density of neurons in the auditory cortex of rats from different groups. Therefore, further study is needed in the future.

## 5. Conclusion

Electroacupuncture at the right TE3 could cause peak latency shift of waves I, III, and V on ABR and waves P0, Nb, and Pb on LLR. This suggests that electroacupuncture at the right TE3 can modulate the neuroplasticity of the central auditory pathway, including the brain stem and primary and secondary auditory cortex. However, this study is just a preliminary study and only observed a phenomenon in healthy rats. Future research will further design a clinical trial to investigate the effect of EA at TE3 on hearing in patients with noise-induced hearing loss, and ABR, MLR, and LLR change are also observed.

## Figures and Tables

**Table 1:** Effect of electroacupuncture at the right TE3 on peak latency and interpeak latency of auditory brainstem response (ABR) recordings.

	Baseline	D3	D5	D8
Peak latency (ms)				
I				
EA	1.007 ± 0.046	1.290 ± 0.024^∗∗^	1.094 ± 0.099	1.005 ± 0.010
Control	1.013 ± 0.041	0.968 ± 0.048	1.005 ± 0.028	0.970 ± 0.057
III				
EA	2.758 ± 0.066	3.475 ± 0.091^∗∗∗^	3.050 ± 0.389	2.785 ± 0.145
Control	2.837 ± 0.202	2.673 ± 0.077	2.675 ± 0.117	2.692 ± 0.257
V				
EA	4.703 ± 0.081	5.880 ± 0.073^∗∗∗^	5.344 ± 0.376	4.738 ± 0.035
Control	4.808 ± 0.124	4.602 ± 0.106	4.457 ± 0.105	4.940 ± 0.539
Interpeak latency (ms)				
I–III				
EA	1.752 ± 0.037	2.185 ± 0.068^∗∗^	1.956 ± 0.292	1.780 ± 0.139
Control	1.823 ± 0.199	1.705 ± 0.037	1.670 ± 0.093	1.722 ± 0.202
III–V				
EA	1.945 ± 0.045	2.405 ± 0.058^∗∗^	2.294 ± 0.155	1.953 ± 0.165
Control	1.972 ± 0.097	1.928 ± 0.099	1.782 ± 0.044^∗^	2.248 ± 0.284
I–V				
EA	3.697 ± 0.070	4.590 ± 0.058^∗∗^	4.250 ± 0.284	3.733 ± 0.043
Control	3.795 ± 0.114	3.633 ± 0.099	3.452 ± 0.085^∗^	3.970 ± 0.485
Latency shift (ms)				
I				
EA	0.000 ± 0.000	0.298 ± 0.033^##^	0.076 ± 0.111	−0.013 ± 0.043
Control	0.000 ± 0.000	−−0.045 ± 0.057	−0.008 ± 0.047	−0.036 ± 0.032
III				
EA	0.000 ± 0.000	0.718 ± 0.038^##^	0.274 ± 0.432	0.030 ± 0.161
Control	0.000 ± 0.000	−0.163 ± 0.130	−0.162 ± 0.316	−0.162 ± 0.374
V				
EA	0.000 ± 0.000	1.160 ± 0.082^##^	0.616 ± 0.433^#^	0.058 ± 0.116
Control	0.000 ± 0.000	−0.207 ± 0.138	−0.352 ± 0.209	0.126 ± 0.544
Interpeak latency shift (ms)				
I–III				
EA	0.000 ± 0.000	0.420 ± 0.041^#^	0.198 ± 0.328	0.043 ± 0.127
Control	0.000 ± 0.000	−0.118 ± 0.177	−0.153 ± 0.292	−0.126 ± 0.343
III–V				
EA	0.000 ± 0.000	0.443 ± 0.069	0.342 ± 0.193^#^	0.028 ± 0.188
Control	0.000 ± 0.000	−0.043 ± 0.139	−0.190 ± 0.110	0.288 ± 0.250
I–V				
EA	0.000 ± 0.000	0.863 ± 0.088^#^	0.540 ± 0.352^#^	0.070 ± 0.100
Control	0.000 ± 0.000	−0.162 ± 0.156	−0.343 ± 0.184	0.162 ± 0.519

Data are presented as mean ± standard deviation (SD); EA: electroacupuncture group; control: control group; D3: third day after baseline recordings, D5: fifth day after baseline recordings; D8: eighth day after baseline recordings: wave I of ABR; III: wave III of ABR; V: wave V of ABR; shift: the value of D3, D5, or D8 minus the baseline value; ^∗^*p* < 0.05, ^∗∗^*p* < 0.01, ^∗∗∗^*p* < 0.001, ^∗∗∗∗^*p* < 0.0001 compared with baseline; ^#^*p* < 0.05, ^##^*p* < 0.01 compared with control.

**Table 2:** Effect of electroacupuncture at the right TE3 on peak latency and interpeak amplitude of late latency response (LLR) recordings.

	Baseline	D3	D5	D8
Peak latency (ms)				
P0				
EA	15.933 ± 1.808	19.889 ± 1.372^∗^	18.133 ± 0.970^∗^	15.600 ± 0.781
Control	14.867 ± 0.825	13.689 ± 1.006	14.556 ± 1.633	15.644 ± 0.953
Na				
EA	23.022 ± 2.244	25.511 ± 1.550	23.156 ± 1.076	22.311 ± 1.439
Control	20.444 ± 1.104	19.889 ± 0.996	20.667 ± 1.225	21.978 ± 1.129
Pa				
EA	38.667 ± 3.953	37.578 ± 3.176	35.044 ± 4.742	33.533 ± 3.816
Control	37.533 ± 2.934	33.533 ± 3.732	37.378 ± 4.356	38.844 ± 3.948
Nb				
EA	56.111 ± 3.056	51.978 ± 5.424	45.378 ± 5.995^∗∗^	42.711 ± 2.739^∗∗∗^
Control	55.311 ± 4.975	52.311 ± 4.042	53.222 ± 7.502	50.356 ± 7.124
Pb				
EA	64.711 ± 1.806	60.422 ± 6.579	56.400 ± 5.334^∗^	50.578 ± 4.065^∗∗∗∗^
Control	69.556 ± 5.895	61.556 ± 5.270	62.889 ± 6.410	57.300 ± 7.161^∗^

Peak latency shift (ms)				
P0				
EA	0.000 ± 0.000	3.956 ± 2.975^##^	2.200 ± 1.889^#^	−0.333 ± 1.949
Control	0.000 ± 0.000	−1.178 ± 1.358	−0.311 ± 1.078	0.778 ± 1.146
Na				
EA	0.000 ± 0.000	2.489 ± 2.965	0.133 ± 2.912	−0.711 ± 1.895
Control	0.000 ± 0.000	−0.556 ± 1.374	0.222 ± 1.185	1.533 ± 1.685
Pa				
EA	0.000 ± 0.000	−1.089 ± 6.018	−3.622 ± 6.686	−5.133 ± 6.993
Control	0.000 ± 0.000	−4.000 ± 4.496	−0.156 ± 6.300	1.311 ± 4.390
Nb				
EA	0.000 ± 0.000	−4.133 ± 4.862	−10.733 ± 6.357	−13.400 ± 4.688
Control	0.000 ± 0.000	−3.000 ± 6.736	−2.089 ± 9.983	−4.956 ± 10.780
Pb				
EA	0.000 ± 0.000	−4.289 ± 6.479	−8.311 ± 5.969	−14.133 ± 4.598
Control	0.000 ± 0.000	−8.000 ± 8.367	−6.667 ± 8.345	−13.300 ± 10.613

Interpeak amplitude (uV)				
P0–Na				
EA	0.779 ± 0.302	0.744 ± 0.683	1.252 ± 0.569	0.815 ± 0.596
Control	0.829 ± 0.098	0.508 ± 0.045	1.285 ± 0.221	0.708 ± 0.393
Na–Pa				
EA	1.765 ± 0.312	2.348 ± 0.680	2.460 ± 0.440^∗^	1.408 ± 0.409^∗^
Control	1.367 ± 0.081	1.145 ± 0.257	1.310 ± 0.793	1.135 ± 0.274
Pa–Nb				
EA	0.733 ± 0.259	0.776 ± 0.225	1.076 ± 0.242	1.345 ± 0.947
Control	0.463 ± 0.133	0.365 ± 0.239	0.558 ± 0.328	0.700 ± 0.456
Nb–Pb				
EA	0.959 ± 0.175	1.074 ± 0.336	1.580 ± 0.648	1.235 ± 0.803
Control	0.968 ± 0.275	0.883 ± 0.205	0.790 ± 0.396	1.148 ± 0.287

Data are presented as mean ± standard deviation (SD); EA: electroacupuncture group; control: control group; D5: fifth day after baseline recordings; D8: eighth day after baseline recordings; P0: wave P0 of LLR; Na: wave Na of LLR; Pa: wave Pa of LLR; Nb: wave Nb of LLR; Pb: wave Pb of LLR; shift: the value of D3, D5, or D8 minus the baseline value; ^∗^*p* < 0.05, ^∗∗^*p* < 0.01, ^∗∗∗^*p* < 0.001 compared with baseline; ^∗∗∗∗^*p* < 0.0001 compared with baseline; ^#^*p* < 0.05, ^##^*p* < 0.01 compared with control.

## Data Availability

The data in this study are available to other researchers upon request.

## Supplementary Materials

Supplementary Materials Supplementary 1. Statistical analysis of wave I, III, and V latency shift of ABR; wave I–III, III–V, and I–V interpeak latency shift of ABR, and wave P0, Na, Pa, Nb, and Pb latency shift of LLR in baseline, D3, D5, and D8.
