# Supplementary material for: Electroacupuncture Promotes Neuroplasticity of Central Auditory Pathway: An Auditory Evoked Potentials Study
Source: Evid Based Complement Alternat Med. 2022 Nov 21;2022:6855775. doi: 10.1155/2022/6855775 (PMC9705092; doi:10.1155/2022/6855775)
Supplement: Supplementary Materials — Supplementary 1. Statistical analysis of wave I, III, and V latency shift of ABR; wave I–III, III–V, and I–V interpeak latency shift of ABR, and wave P0, Na, Pa, Nb, and Pb latency shift of LLR in baseline, D3, D5, and D8. [file 6855775.f1.pdf]

	EA						Control					
B	0	0	0	0	0	0	0	0	0	0	0	0
D3	0.32	0.25		0.32	0.3		0.03	-0.04	-0.12	0	-0.04	-0.1
D5		-0.08	0.18	0.15	0	0.13	-0.02	0.05	-0.05	-0.05	0.05	-0.03
D8	0.05	-0.03	-0.02			-0.05	-0.07	-0.02	0	-0.07	-0.02	

Compare each cell mean with the other cell mean in that row

Number of families	1
Number of comparisons per family	4
Alpha	0.05

Bonferroni's multiple comparisons test	Mean Diff.	95.00% CI of diff.	Below threshold?	Summary	Adjusted P Value
EA - Control					
B	0				
D3	0.3425	0.2322 to 0.4528	Yes	**	0.0019
D5	0.08433	-0.2140 to 0.3827	No	ns	>0.9999
D8	0.0235	-0.3064 to 0.3534	No	ns	>0.9999

Test details	Mean 1	Mean 2	Mean Diff.	SE of diff.	N1	N2	t	DF
EA - Control								
B	0	0	0	0	6	6		
D3	0.2975	-0.045	0.3425	0.02045	4	6	16.74	3
D5	0.076	-0.008333	0.08433	0.06914	5	6	1.22	4
D8	-0.0125	-0.036	0.0235	0.03724	4	5	0.6311	2

III	EA						Control					
B	0	0	0	0	0	0	0	0	0	0	0	0
D3	0.75	0.69		0.68	0.75		-0.33	-0.07	-0.1	-0.33	-0.07	-0.08
D5		-0.2	0.67	0.45	-0.18	0.63	-0.55	0.1	-0.02	-0.58	0.08	0
D8	0.23	0.09	-0.08			-0.12	-0.55	-0.07	0.38	-0.48	-0.09	

Compare each cell mean with the other cell mean in that row

Number of families	1
Number of comparisons per family	4
Alpha	0.05

Bonferroni's multiple comparisons test	Mean Diff.	95.00% CI of diff.	Below threshold?	Summary	Adjusted P Value
EA - Control					
B	0				
D3	0.8808	0.4926 to 1.269	Yes	**	0.0047
D5	0.4357	-0.6884 to 1.560	No	ns	0.6791
D8	0.192	-2.419 to 2.803	No	ns	>0.9999

Test details	Mean 1	Mean 2	Mean Diff.	SE of diff.	N1	N2	t	DF
EA - Control								
B	0	0	0	0	6	6		
D3	0.7175	-0.1633	0.8808	0.072	4	6	12.23	3
D5	0.274	-0.1617	0.4357	0.2605	5	6	1.672	4
D8	0.03	-0.162	0.192	0.2947	4	5	0.6516	2

V	EA						Control					
B	0	0	0	0	0	0	0	0	0	0	0	0
D3	1.27	1.17		1.08	1.12		-0.27	-0.28	-0.23	-0.3	0.07	-0.23
D5		0.2	1.08	0.45	0.27	1.08	-0.59	-0.17	-0.25	-0.64	-0.18	-0.28
D8	0.17	-0.1	0.05			0.11	-0.27	-0.05	1.07	-0.19	0.07	

Compare each cell mean with the other cell mean in that row

Number of families	1
Number of comparisons per family	4
Alpha	0.05

Bonferroni's multiple comparisons test	Mean Diff.	95.00% CI of diff.	Below threshold?	Summary	Adjusted P Value
EA - Control					
B	0				
D3	1.367	0.8399 to 1.893	Yes	**	0.0032
D5	0.9677	0.07910 to 1.856	Yes	*	0.0373
D8	-0.0685	-3.275 to 3.138	No	ns	>0.9999

Test details	Mean 1	Mean 2	Mean Diff.	SE of diff.	N1	N2	t	DF
EA - Control								
B	0	0	0	0	6	6		
D3	1.16	-0.2067	1.367	0.0977	4	6	13.99	3
D5	0.616	-0.3517	0.9677	0.2059	5	6	4.699	4
D8	0.0575	0.126	-0.0685	0.3619	4	5	0.1893	2

I-III	EA						Control					
B	0	0	0	0	0	0	0	0	0	0	0	0
D3	0.43	0.44		0.36	0.45		-0.36	-0.03	0.02	-0.33	-0.03	0.02
D5		-0.12	0.49	0.3	-0.18	0.5	-0.53	0.05	0.03	-0.53	0.03	0.03
D8	0.18	0.12	-0.06			-0.07	-0.48	-0.05	0.38	-0.41	-0.07	

Compare each cell mean with the other cell mean in that row

Number of families	1
Number of comparisons per family	4
Alpha	0.05

Bonferroni's multiple comparisons test	Mean Diff.	95.00% CI of diff.	Below threshold?	Summary	Adjusted P Value
EA - Control					
B	0				
D3	0.5383	0.1017 to 0.9749	Yes	*	0.0277
D5	0.3513	-0.4962 to 1.199	No	ns	0.5928
D8	0.1685	-2.156 to 2.493	No	ns	>0.9999

Test details	Mean 1	Mean 2	Mean Diff.	SE of diff.	N1	N2	t	DF
EA - Control								
B	0	0	0	0	6	6		
D3	0.42	-0.1183	0.5383	0.08097	4	6	6.648	3
D5	0.198	-0.1533	0.3513	0.1964	5	6	1.789	4
D8	0.0425	-0.126	0.1685	0.2624	4	5	0.6422	2

III-V	EA						Control					
B	0	0	0	0	0	0	0	0	0	0	0	0
D3	0.52	0.48		0.4	0.37		0.06	-0.21	-0.13	0.03	0.14	-0.15
D5		0.4	0.41	4.4409E-16	0.45	0.45	-0.04	-0.27	-0.23	-0.06	-0.26	-0.28
D8	-0.06	-0.19	0.13			0.23	0.28	0.02	0.69	0.29	0.16	

Compare each cell mean with the other cell mean in that row

Number of families	1
Number of comparisons per family	4
Alpha	0.05

Bonferroni's multiple comparisons test	Mean Diff.	95.00% CI of diff.	Below threshold?	Summary	Adjusted P Value
EA - Control					
B	0				
D3	0.4858	-0.008877 to 0.9805	No	ns	0.0526
D5	0.532	0.005905 to 1.058	Yes	*	0.0481
D8	-0.2605	-1.194 to 0.6725	No	ns	0.5274

Test details	Mean 1	Mean 2	Mean Diff.	SE of diff.	N1	N2	t	DF
EA - Control								
B	0	0	0	0	6	6		
D3	0.4425	-0.04333	0.4858	0.09175	4	6	5.295	3
D5	0.342	-0.19	0.532	0.1219	5	6	4.363	4
D8	0.0275	0.288	-0.2605	0.1053	4	5	2.474	2

I-V	EA						Control					
B	0	0	0	0	0	0	0	0	0	0	0	0
D3	0.95	0.92		0.76	0.82		-0.3	-0.24	-0.11	-0.3	0.11	-0.13
D5		0.28	0.9	0.3	0.27	0.95	-0.57	-0.22	-0.2	-0.59	-0.23	-0.25
D8	0.12	-0.07	0.07			0.16	-0.2	-0.03	1.07	-0.12	0.09	

Compare each cell mean with the other cell mean in that row

Number of families	1
Number of comparisons per family	4
Alpha	0.05

Bonferroni's multiple comparisons test	Mean Diff.	95.00% CI of diff.	Below threshold?	Summary	Adjusted P Value
EA - Control					
B	0				
D3	1.024	0.4393 to 1.609	Yes	*	0.0101
D5	0.8833	0.2698 to 1.497	Yes	*	0.0137
D8	-0.092	-3.059 to 2.875	No	ns	>0.9999

Test details	Mean 1	Mean 2	Mean Diff.	SE of diff.	N1	N2	t	DF
EA - Control								
B	0	0	0	0	6	6		
D3	0.8625	-0.1617	1.024	0.1085	4	6	9.442	3
D5	0.54	-0.3433	0.8833	0.1422	5	6	6.212	4
D8	0.07	0.162	-0.092	0.3349	4	5	0.2747	2

P0	EA									Control								
B	0	0	0	0	0	0	0	0	0	0	0	0	0	0	0	0	0	0
D3	3.8	5.8	2.6	4.8	9.4	2.2	3.6	5	-1.6	0.6	-2	-2.2	-3.2	-1.8	-0.8	1	-0.6	-1.6
D5	2.6	3	3.2	3.8	4.2	2	0.2	2.6	-1.8	0	-0.4	-1.2	2	-1.6	-0.6	0.4	-0.2	-1.2
D8	0.4	-1.2	-1.4	1.2	2.4	-0.8	0	0.8	-4.4	2.2	1.6	2	-0.4	0	1.2	0	-1	1.4

Compare each cell mean with the other cell mean in that row

Number of families	1
Number of comparisons per family	4
Alpha	0.05

Bonferroni's multiple comparisons test	Mean Diff.	95.00% CI of diff.	Below threshold?	Summary	Adjusted P Value
EA - Control					
B	0				
D3	5.133	1.895 to 8.372	Yes	**	0.0024
D5	2.511	0.4036 to 4.619	Yes	*	0.0173
D8	-1.111	-3.296 to 1.074	No	ns	0.6576

Test details	Mean 1	Mean 2	Mean Diff.	SE of diff.	N1	N2	t	DF
EA - Control								
B	0	0	0	0	9	9		
D3	3.956	-1.178	5.133	1.09	9	9	4.71	11.2
D5	2.2	-0.3111	2.511	0.725	9	9	3.463	12.71
D8	-0.3333	0.7778	-1.111	0.7538	9	9	1.474	12.94

Na	EA									Control								
B	0	0	0	0	0	0	0	0	0	0	0	0	0	0	0	0	0	0
D3	-2.4	5.6	2.8	1.2	6.8	0	2.4	5.2	0.8	-0.2	-2.2	-2.2	1.2	0.6	0.6	0.4	-2.2	-1
D5	-3.2	1.4	2.4	-1.8	4.8	-0.6	0	2.4	-4.2	1.4	0	-1.4	2	0.8	0.4	0.8	-0.6	-1.4
D8	-0.8	-1.6	0.2	0	3	-1.6	-2	0.2	-3.8	2	2.4	0.4	1.4	1.8	5	-0.4	-0.6	1.8

Compare each cell mean with the other cell mean in that row

Number of families	1
Number of comparisons per family	4
Alpha	0.05

Bonferroni's multiple comparisons test	Mean Diff.	95.00% CI of diff.	Below threshold?	Summary	Adjusted P Value
EA - Control					
B	0				
D3	3.044	-0.1871 to 6.276	No	ns	0.0682
D5	-0.08889	-3.236 to 3.059	No	ns	>0.9999
D8	-2.244	-4.626 to 0.1375	No	ns	0.0697

Test details	Mean 1	Mean 2	Mean Diff.	SE of diff.	N1	N2	t	DF
EA - Control								
B	0	0	0	0	9	9		
D3	2.489	-0.5556	3.044	1.089	9	9	2.795	11.28
D5	0.1333	0.2222	-0.08889	1.048	9	9	0.08482	10.58
D8	-0.7111	1.533	-2.244	0.8453	9	9	2.655	15.78

Pa	EA									Control								
B	0	0	0	0	0	0	0	0	0	0	0	0	0	0	0	0	0	0
D3	-5.8	-2.6	-7.6	-2.2	4.6	-3.6	-1.2	12.2	-3.6	4.6	-10	-4.8	-2	-6.8	-4.8	-6.8	-6.4	1
D5	-8	5	-8.6	-6	7	-7.4	-4	1.8	-12.4	1.8	-5.4	8.4	-8.6	0.4	-2.4	-6.8	2	9.2
D8	-9.8	-9.4	-10	-1.4	1	-0.8	-9.6	7.6	-13.8	-7.2	-1	0.6	0.8	1.2	0.2	3	5.6	8.6

Compare each cell mean with the other cell mean in that row

Number of families	1
Number of comparisons per family	4
Alpha	0.05

Bonferroni's multiple comparisons test	Mean Diff.	95.00% CI of diff.	Below threshold?	Summary	Adjusted P Value
EA - Control					
B	0				
D3	2.911	-4.204 to 10.03	No	ns	>0.9999
D5	-3.467	-12.08 to 5.151	No	ns	>0.9999
D8	-6.444	-14.37 to 1.484	No	ns	0.1407

Test details	Mean 1	Mean 2	Mean Diff.	SE of diff.	N1	N2	t	DF
EA - Control								
B	0	0	0	0	9	9		
D3	-1.089	-4	2.911	2.504	9	9	1.163	14.81
D5	-3.622	-0.1556	-3.467	3.062	9	9	1.132	15.94
D8	-5.133	1.311	-6.444	2.752	9	9	2.342	13.46

Nb	EA									Control								
B	0	0	0	0	0	0	0	0	0	0	0	0	0	0	0	0	0	0
D3	-6.8	-11.4	-1	-8.4	-2	-5.2	-7.2	3	1.8	-9.4	-13	1.4	4.6	-2	-5.2	-1.8	-8.8	7.2
D5	-8.2	1.4	-14.4	-9.2	-8.2	-19	-7	-13.8	-18.2	-11.6	-11	11	-12.4	2.6	-11.4	0.6	0.4	13
D8	-12.6	-15.8	-19.6	-11.4	-7.4	-16.4	-12.4	-6.2	-18.8	-23.8	-11	-6.4	0	-3.8	-14.2	4.2	-3	13.4

Compare each cell mean with the other cell mean in that row

Number of families	1
Number of comparisons per family	4
Alpha	0.05

Bonferroni's multiple comparisons test	Mean Diff.	95.00% CI of diff.	Below threshold?	Summary	Adjusted P Value
EA - Control					
B	0				
D3	-1.133	-9.021 to 6.754	No	ns	>0.9999
D5	-8.644	-19.99 to 2.706	No	ns	0.1857
D8	-8.444	-20.14 to 3.252	No	ns	0.2174

Test details	Mean 1	Mean 2	Mean Diff.	SE of diff.	N1	N2	t	DF
EA - Control								
B	0	0	0	0	9	9		
D3	-4.133	-3	-1.133	2.769	9	9	0.4093	14.56
D5	-10.73	-2.089	-8.644	3.945	9	9	2.191	13.57
D8	-13.4	-4.956	-8.444	3.918	9	9	2.155	10.92

Pb	EA									Control								
B	0	0	0	0	0	0	0	0	0	0	0	0	0	0	0	0	0	0
D3	-16	-8.6	1.6	-8.6	-4.2	-3.4	-5.8	0.8	5.6	-14.6	-22.4	-5.2	-3.2	-6.2	-7.2	-3.2	-15.8	5.8
D5	-14.8	-1	-14.4	-10.6	-13.4	-10	-2.4	0.8	-9	-12.2	-17.6	-1.2	-7	-1.6	-11.4	0.6	-16.4	6.8
D8	-18.8	-15.4	-13.6	-8.4	-12.4	-17	-7	-13.4	-21.2	-26	-23.2	-18.8	0.4	-1.8	-16.4	-1.6	-19	

Compare each cell mean with the other cell mean in that row

Number of families	1
Number of comparisons per family	4
Alpha	0.05

Bonferroni's multiple comparisons test	Mean Diff.	95.00% CI of diff.	Below threshold?	Summary	Adjusted P Value
EA - Control					
B	0				
D3	3.711	-6.289 to 13.71	No	ns	>0.9999
D5	-1.644	-11.39 to 8.103	No	ns	>0.9999
D8	-0.8333	-13.35 to 11.68	No	ns	>0.9999

Test details	Mean 1	Mean 2	Mean Diff.	SE of diff.	N1	N2	t	DF
EA - Control								
B	0	0	0	0	9	9		
D3	-4.289	-8	3.711	3.527	9	9	1.052	15.06
D5	-8.311	-6.667	-1.644	3.42	9	9	0.4808	14.49
D8	-14.13	-13.3	-0.8333	4.053	9	8	0.2056	9.304
